# Supplementary material for: Retroviral Replicating Vector Toca 511 (Vocimagene Amiretrorepvec) for Prodrug Activator Gene Therapy of Lung Cancer
Source: Cancers (Basel). 2022 Nov 25;14(23):5820. doi: 10.3390/cancers14235820 (PMC9736610; doi:10.3390/cancers14235820)
Supplement: Supplementary file 1 [file cancers-14-05820-s001.zip › cancers-2044575-supplementary.pdf]

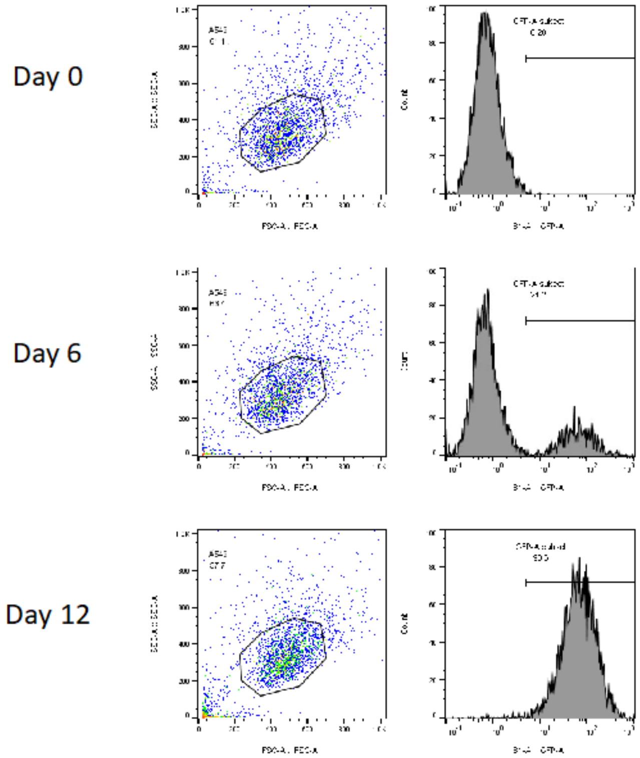

Figure S1. The cytofluorimetric data in A549 In A549 cells, the percentage of GFP-positive cells progressively increased over time and reached 80% within 12 days after inoculation.

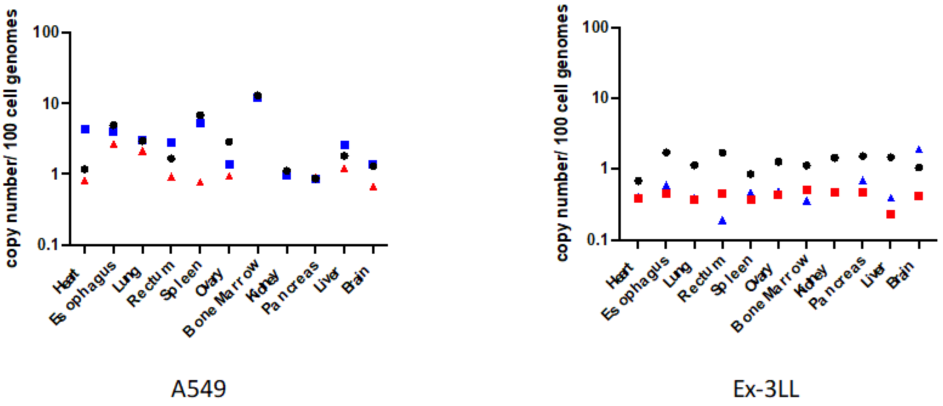

Figure S2. The logarithmic data systemic biodistribution of Toca 511 in subcutaneous lung cancer models. In the subcutaneous A549 human lung cancer xenograft model, RRV signaling was expressed primarily in the spleen and bone marrow, whereas in the Ex-3LL mouse lung cancer model, there was minimal systemic biodistribution of RRV in all tissues examined.

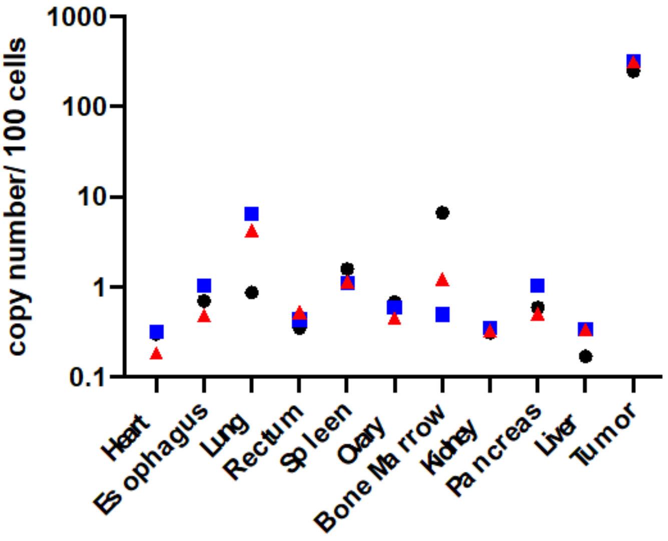

Figure S3. The logarithmic data systemic biodistribution of Toca 511 in orthotopic human lung cancer model.Systemic biodistribution of RRV after intrathoracic injection was examined by qPCR of genomic DNA. As expected, high levels of RRV signal were observed in tumor tissues, while only low levels of RRV were detected in normal tissues.

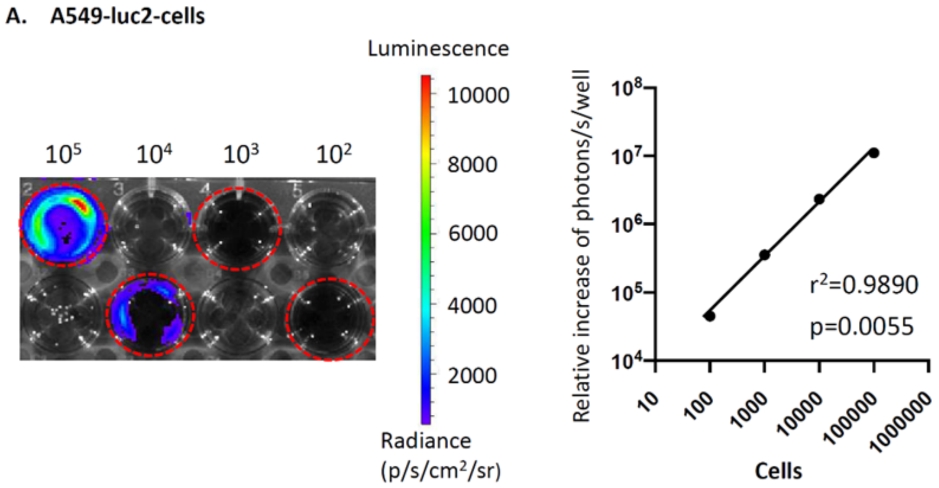

Figure S4. (A) A549-luc2 and (B) Ex-3LL-luc2 cell suspensions were serially diluted and plated into wells (105, 104, 103, 102 cells per well, as indicated), incubated with Luciferin substrate, and analyzed for bioluminescence by IVIS optical imaging. Luminescent signal intensity values (expressed as photons/s/well) were determined according to the radiance scales as shown, and plotted against cell numbers. For both A549-luc2 and Ex-3LL-luc2 cells, a strong positive correlation between cell numbers and luminescence was observed (correlation coefficients (r2) = 0.99 for both cell lines).
